# Exploring the Inherent Heterogeneity of Vaccine Hesitancy: A Study of a Childhood-Vaccine-Hesitant Population

**DOI:** 10.3390/vaccines12080839

**Published:** 2024-07-25

**Authors:** Monika Lamot, Andrej Kirbiš, Mitja Vrdelja

**Affiliations:** 1 Department of Sociology, Faculty of Arts, University of Maribor, 2000 Maribor, Slovenia; 2 National Institute of Public Health, 1000 Ljubljana, Slovenia; mitja.vrdelja@nijz.si

**Keywords:** vaccine hesitancy, heterogeneity, latent profile analysis, Slovenia, complementary and alternative medicine (CAM), conspiracy theories, trust in healthcare system

## Abstract

Vaccine hesitancy and its determinants have been previously widely researched. Vaccine hesitancy has been defined as a continuum of attitudes, ranging from accepting vaccines with doubts to rejecting them. The present study aims to explore the heterogeneity of a childhood-vaccine-hesitant group by using a person-oriented approach–latent profile analysis. A non-representative cross-sectional sample of vaccine-hesitant Slovenians (N = 421, M_age_ = 35.21, 82.9% women) was used to identify differences based on their reliance on personal research (“self” researching instead of relying on science), overconfidence in knowledge, endorsement of conspiracy theories, complementary and alternative medicine, and trust in the healthcare system. The analysis revealed three profiles of vaccine-hesitant individuals. The most hesitant profile—vaccine rejecting—expressed the greatest reliance on personal research, expressed the highest endorsement of conspiracy theories and complementary and alternative medicine, showed moderate overconfidence in their knowledge, and expressed the highest levels of distrust in the healthcare system. We further found differences in sociodemographic structure and that the identified profiles differed in their attitudes regarding MMR, HPV, and Seasonal Influenza vaccinations. The present study demonstrates the heterogeneity of the vaccine-hesitant community and offers insights into some of the traits, which are crucial for designing pro-vaccine campaigns.

## 1. Introduction

Vaccine hesitancy has been defined by prior research as a continuum of attitudes [1], yet the individuals exhibiting vaccine-hesitant attitudes are often treated as a homogenous group [2,3]. However, recent studies challenge this perspective, showing that treating vaccine-hesitant people as a homogenous group, i.e., merely comparing vaccine hesitant groups to non-vaccine hesitant groups, may limit the full understanding of the complexities within vaccine-hesitant individuals [4,5,6]. Recent work by Howard [4] and Zhou et al. [5] highlights the importance of a nuanced understanding of members of groups which express reservations or fully oppose vaccinations. Specifically, Howard [4] studied a diverse sample from several countries, including the United States, Portugal, Poland, United Kingdom, Mexico, and Italy. The sample consisted of both vaccine-hesitant and non-hesitant individuals, and eight distinct profiles were identified. Amongst vaccine-hesitant individuals, a profile named ‘Distrusting’ emerged, characterized by concerns about vaccine safety and efficacy, as well as politically conservative views, which demonstrated the least willingness to vaccinate. Zhou et al. [5] classified vaccine-hesitant individuals from the U.S. into five profiles based on their psychological characteristics, such as fear and disgust, further underscoring the group’s complexity.

The present study aims to build on these insightful works by examining the within-group heterogeneity of vaccine-hesitant individuals and investigating whether they differ based on the depth (i.e., intensity) of their hesitancy, specific attitudes about vaccines and vaccination, attitudes towards specific vaccines (Measles, Mumps, and Rubella (MMR), human papillomavirus (HPV), and Seasonal Influenza), and sociodemographic characteristics. In addition, there is a lack of studies on vaccine-hesitant groups from East–Central European countries, as most studies focus on Western countries. Examining this populace is particularly critical, as several studies show that East and Central Europeans express some of the highest levels of vaccine hesitancy worldwide [7,8].

## 2. Predictors of Vaccine Hesitancy

The previous literature has explored various predictors of vaccine hesitancy, including positive attitudes towards complementary and alternative medicine [9,10], distrust in the healthcare system [11,12], endorsement of conspiracy theories [13,14,15], engagement in personal research (information-seeking behavior on the Internet) [16,17], and overconfidence in one’s knowledge [18]. While these studies offer valuable insights into the phenomenon of vaccine hesitancy, much less is known about the different intensities of these beliefs within the vaccine-hesitant population.

In the present study, we examined the predictors of vaccine hesitancy as latent indicators upon which vaccine-hesitant individuals were classified into subgroups. The following research question was posited:

*Research Question 1*: Do distinct profiles of vaccine hesitant individuals exist that vary qualitatively and quantitatively in their endorsement of complementary and alternative medicine, conspiracy theories, trust in the healthcare system, engagement in “self” researching information, and overconfidence in their knowledge?

### 2.1. Sociodemographic Characteristics of Vaccine Hesitant Individuals 

Research has identified several sociodemographic determinants of vaccine hesitancy, including gender [19,20], age [21,22], lower income [3,23,24], lower education [25,26], and political orientation [27,28,29]. It has also been established that determinants of vaccine hesitancy vary across countries [7,30], further confirming the complexities of the vaccine-hesitant population. 

The importance of the need for a fine-grained investigation of vaccine-hesitant groups and their traits was demonstrated by recent studies employing latent profile analysis (LPA), which gave insights into the underlying sociodemographic characteristics among vaccine-hesitant individuals. For instance, work by Hornsey et al. [31], Gravelle et al. [32], Lamot et al. [6], and Howard [4] revealed differences in sociodemographic structures within the vaccine-hesitant population, demonstrating the potential value of a more detailed, subgroup-level analysis. Gravelle et al. [32], for instance, found that the youngest people were the most polarized about vaccines, with strong feelings for or against vaccination. In addition, individuals who were extremely conservative were more likely to be vaccine hesitant or anti-vaccine.

*Research Question 2*: Do sociodemographic determinants (gender, age, education, income, and political orientation) predict the profile membership of vaccine-hesitant individuals?

### 2.2. Do Vaccine Hesitant Individuals Differ on Their Stance on Vaccination and Specific Vaccines?

Research on general populations demonstrates a clear association between vaccine hesitancy and concerns about vaccine safety [33,34,35], the perceived efficacy of vaccines for preventing infectious diseases [36,37,38], and the perceived role of vaccination for one’s personal and public health [39].

However, less attention has been paid to such concerns within vaccine-hesitant groups. For example, Howard [4] demonstrates that within-group differences do exist; specifically, he found that the most vaccine-hesitant profile differed in terms of their mean score regarding vaccine safety in comparison to the other identified profiles. This underscores the complexity of vaccine attitudes and concerns within vaccine-hesitant individuals.

While recent research has primarily focused on attitudes towards the COVID-19 vaccine due to the most recent pandemic [40,41,42], the pre-pandemic literature has also examined vaccine hesitancy related to the MMR, HPV, and Seasonal Influenza vaccines [43,44]. Weiss et al. [45] conducted a Latent Class Analysis of parents of young children and examined their attitudes towards MMR vaccination. The analysis identified three classes, two of which expressed vaccine-hesitant and anti-vaccine sentiments. The “hesitant” group doubted that vaccination is necessary (i.e., did not perceive the three diseases as threatening). On the other hand, the third identified class (the vaccine-rejecting one) more likely agreed that immunization is an artificial intrusion into the natural immune system and therefore wanted to vaccinate their children only if necessary.

*Research Question 3*: Do identified profiles differ in vaccine-related concerns (vaccine safety, efficacy, and importance)?

*Research Question 4*: Do identified profiles differ in attitudes towards specific vaccines (against MMR, HPV, and Seasonal Influenza)?

### 2.3. The Role of Information Sources in Understanding Vaccine Hesitancy

Finally, the present study explored the importance of information sources in shaping vaccine attitudes among the vaccine-hesitant individuals. The sources where individuals seek information about vaccines can greatly influence their perceptions and decisions, ranging from mainstream media and healthcare providers to social networks and Internet searches [46,47,48]. Charron et al. [46] found that greater vaccination intention was found among individuals who obtained information about vaccines from healthcare professionals, while vaccine hesitancy was associated with obtaining information from the Internet or from relatives. Furthermore, Reno et al. [48] found that vaccine-hesitant individuals were less likely to obtain information from the mainstream media and more likely to obtain information from social media. However, there remains a gap in understanding how obtaining information about vaccines from different sources differs among the subgroups of the vaccine-hesitant population.

*Research Question 5*: Do identified profiles differ in the importance they give to different information sources about vaccines?

## 3. Methods

### 3.1. Data Collection

The data were collected in November 2019 using the web-based survey platform 1ka (https://www.1ka.si/ (accessed on 24 July 2024). A snowball sample strategy was used that involved the social media networks Facebook and Twitter. According to Thornton et al. [49] and King et al. [50], recruitment via social media is especially beneficial for addressing difficult-to-reach communities. We thus used this sampling strategy to increase the number of vaccine-hesitant people in our sample. However, because of the nature of this sampling technique, the sample does not represent the entire Slovenian vaccine-hesitant population.

The data gathering procedure adhered strictly to the standards of the Helsinki Declaration. Before they began filling out the questionnaire, respondents were given a brief overview of the study and its aims. They were also informed that their participation was anonymous and voluntary and that they could opt out at any time. After reading this information, participants proceeded to the questionnaire, confirming their informed consent.

### 3.2. Participants

A total of 661 individuals from Slovenia completed the survey. Because we focused on vaccine-hesitant individuals, we excluded participants who expressed positive attitudes toward vaccines. This exclusion was determined based on their response to a question about their attitudes toward vaccines. Individuals who responded with “All recommended childhood vaccines should be given to all children who have no contraindications; I have no doubts about them” were removed from the sample, as their response did not indicate any hesitancy towards childhood vaccines. Thus, our final sample included 421 vaccine-hesitant individuals, all of whom were over 18 years old. The median age of the sample was 35.21 years. The sample was predominantly composed of women (82.9%).

### 3.3. Measures

#### Latent Indicators

Several attitudes were measured to identify characteristics of vaccine-hesitant groups. Attitudes towards complementary and alternative medicine were assessed through five items from the Holistic Complementary and Alternative Health Questionnaire (HCAMQ), for example, “It’s always worth trying complementary and alternative medicine before going to the doctor” [51]. The respondents gave their answers on a 5-point Likert scale, with options ranging from strongly disagree (1) to strongly agree (5). The scale demonstrated great internal consistency (Cronbach’s alpha = 0.82).

Endorsement of conspiracy theories was measured with several items. We used two items from the Generic Conspiracist Beliefs questionnaire, “Some important societal events were the result of the actions of a small group secretly manipulating world events” and “The spread of some viruses and diseases is the result of deliberate, covert efforts by certain organizations” [52]. Next, the statement “The physical traces that airplanes leave in the sky (i.e., chemtrails) are chemical traces or chemical weapons” was self-developed. We also added the item “Vaccination programs are profit-motivated by the pharmaceutical industry” due to some studies indicating a link between anti-vaccination attitudes and beliefs about the pharmaceutical lobbying of vaccination programs [53]. Responses were measured on a 5-point Likert scale (1 = strongly disagree, 5 = strongly agree). The four-item construct demonstrated high internal reliability (Cronbach’s alpha = 0.82).

The reliance on personal research was tapped with the statement “Instead of relying on science and scientists, it is better if an individual informs themselves before making important decisions.” Responses were collected on a 4-point Likert scale (1 = strongly disagree, 4 = strongly agree). Overconfidence was evaluated through the following two statements: “I think my knowledge in general is comparable to the knowledge of doctors”, and “I think my knowledge in general is comparable to the knowledge of scientists”. Participants responded on a 5-point Likert scale (1 = strongly disagree, 5 = strongly agree). The two variables were combined into a single item based on the median score (Spearman–Brown’s coefficient = 0.86, *p* < 0.001).

### 3.4. Outcomes

The intensity of vaccine hesitancy was measured with the question “Which of the following statements best describes your attitudes towards vaccination?” Possible answers were (1) all recommended children’s vaccines should be given to all children who have no contraindications; however, I have some reservations about them, (2) most recommended children’s vaccines need to be given to children who have no contraindications, but not in all cases and/or not for all vaccines that are in the vaccination program, (3) some recommended children’s vaccines need to be given to children who have no contraindications, but in most cases and/or not for the majority of vaccines that are in the vaccination program, and (4) children should not be given any of the recommended vaccines [54].

We measured general attitudes toward vaccination with three items, “In general, I think vaccines are effective”, “In general, I think vaccines are safe”, and “Vaccines are important for the health of children”. The items were measured on a 5-point Likert scale (1 = strongly disagree, 5 = strongly agree).

Attitudes towards specific vaccines were also examined. For the MMR vaccine, we used the following statement “Scientific evidence shows that there is no link between the MMR vaccine and autism” [55]. Attitudes towards there HPV vaccine were tapped with “The current HPV vaccine can prevent the onset of cervical cancer” [56]. Attitudes towards Seasonal Influenza vaccines were measured with the following statement: “I find the seasonal flu vaccine to be very safe” [25]. The respondents gave their answers on a 5-point Likert scale, ranging from strongly disagree (1) to strongly agree (5).

Finally, the importance of information sources about vaccines was measured on a 5-point scale (1 = not important at all; 5 = very important). Respondents rated the importance of the following information sources: doctor, family, friends, the National Institute of Public Health, forums and websites, TV, newspapers, alternative healers (such as homeopath and chiropractic), and social media.

### 3.5. Sociodemographic Predictors

The sociodemographic variables included in the analysis were gender (1 = male, 2 = female), age (in years), income (respondents wrote their income in EUR), education (1 = uncompleted elementary education; 11 = PhD), and political orientation (0 = left; 10 = right).

### 3.6. Statistical Analyses

Latent profile analysis (LPA) was employed in our analyses. LPA is a statistical method employed for identifying “latent” subgroups within a larger population based on their characteristics. It enhances better understanding of the underlying structure of a population and how different determinants may be related to group membership [57]. The present paper used Mplus 8.3 for conducting LPA. Deciding the profile solution (i.e., the number of profiles) included several model-fit statistics. Standard indicators used in LPA include the Bayesian Information Criterion (BIC), Sample-adjusted Bayesian Information Criterion (SABIC), Akaike’s Information Criterion (AIC), Bootstrap Likelihood Ratio test (BLRT), Lo–Mendell–Rubin test (LMR), entropy, and posterior classification [58]. In this study, special consideration was given to LMR and BLRT, which compare the k_0_ model with the k_−1_ model. A significant value of the BLRT and LMR tests implies that the k_0_ solution is superior to the k_−1_ solution. BIC and SABIC were also of particular interest—the lower values of these criteria suggest a better model fit, as AIC and entropy have often shown poor performance in selecting the correct number of classes [59]. Other criteria, such as smallest profile size and interpretability, were also considered when determining the number of profiles. As Ferguson et al. [57] emphasize, profiles comprising less than 5% of the sample might be misleading. Moreover, considerations were also made in terms of interpretability, especially in evaluating if an additional profile offers new and significant perspectives.

After deciding on the number of profiles, the analysis was carried out in SPSS 28. Using class probabilities, we examined if there were quantitative differences among the profiles in relation to the five latent variables. Predictors of profile membership were analyzed using multinomial regression, while differences in vaccine attitudes between profiles were examined with analysis of variance (ANOVA).

## 4. Results

Model fit statistics are presented in Table 1. The Bootstrap Likelihood Ratio test (BLRT) indicated a five-profile solution, while the Lo–Mendell–Rubin (LMR) test suggested a four-profile solution. When comparing the three- and four-profile solutions, we found that the Akaike Information Criterion (AIC), Bayesian Information Criterion (BIC), and Sample-Size Adjusted Bayesian Information Criterion (SA-BIC) did not decrease substantially. Moreover, the three-profile solution yielded both higher entropy and a larger proportion of the smallest class. Furthermore, we examined the interpretability of the profiles and found that the additional profile in the four-profile solution did not differ substantially. Therefore, considering all model fit indices, the principles of parsimony, and interpretability, we opted for a three-profile solution.

ANOVA (Table 2) revealed statistically significant differences between profiles in endorsement of complementary and alternative medicine (CAM) and conspiracy theories, preference for self-research, trust in the healthcare system, and tendency toward overconfidence in own knowledge. Post hoc comparisons using Tukey’s test, required because of the unequal profile sizes, showed that all pairwise comparisons were significant at the *p* < 0.001 level for both CAM and endorsement of conspiracy theories. Specifically, profile 1 showed a statistically significantly higher tendency to endorse CAM compared to profile 2, although it was lower than profile 3. Regarding conspiracy theories, profile 3 was more likely to uphold such theories than either profile 1 or profile 2, while the latter showed less positive attitudes toward conspiracies compared to profile 1. 

Further pairwise comparisons were examined using the Games–Howell post hoc test to account for unequal group sizes and violation of the assumption of homogeneity of variances when examining differences in personal research, trust in the healthcare system, and overconfidence in knowledge. It was found that profile 2 was less inclined toward personal research compared to profiles 1 and 3, with profile 3 showing the greatest tendency among the three. In addition, profile 3 demonstrated the most negative attitudes toward the healthcare system compared to profiles 1 and 2, with profile 1 showing a significantly more positive attitudes than profile 2; all differences reached the *p* < 0.001 level. Finally, regarding overconfidence in knowledge, profile 3 showed significantly greater overconfidence in own knowledge than profiles 1 or 2 (*p* < 0.001), while no significant difference was found between profiles 1 and 2. 

**Figure 1:**
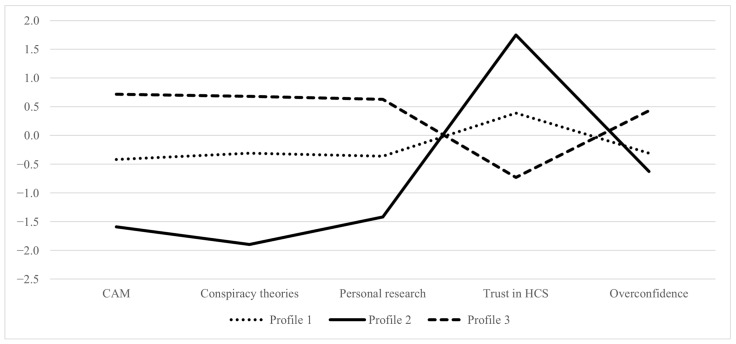
Z-standardized values.

The three extracted profiles thus demonstrated unique patterns across CAM and conspiracy theories endorsement, personal research, trust in the healthcare system, and overconfidence in knowledge. The standardized values of the indicator variables for each extracted profile are shown in Figure 1. A value of zero represents the sample mean, whereas negative values represent scores below the sample mean and positive values represent scores above the sample mean. The magnitude of these numbers shows the distance from the sample mean in standard deviation units.

Profile 1, labeled Skeptics, consisted of 184 individuals who demonstrated moderate trust in the healthcare system, low endorsement of both CAM and conspiracy theories, lower reliance on personal research, and low overconfidence in knowledge. The second profile (Conventionalists, *n* = 41) revealed a similar pattern to Skeptics, However, the two notably differ in the magnitude of their attitudes. Specifically, profile 2 exhibited high trust in the healthcare system and the lowest endorse=ment of CAM and conspiracies, personal research, and overconfidence. Despite being the smallest profile, it is noteworthy that it displayed the highest trust in the healthcare system among the three vaccine-hesitant groups. Lastly, profile 3 (Self-directed researchers, *n* = 196) was the largest profile and presented a very different pattern from the previous two. Profile 3 revealed high endorsement of CAM and conspiracy theories, a tendency towards personal research, distrust in the healthcare system, and moderate overconfidence in knowledge. 

Next, we were interested in whether gender, age, income, education, and political orientation predict profile membership (Table 3). The multinomial logistic regression model was statistically significant, outperforming the null model (χ^2^ (10) = 47,321, Nagelkerke *R*^2^ = 0.14, *p* < 0.001). For profile 1 (Skeptics) membership, age, income, education, and gender were significant predictors. Those who were younger (*b* = −0.03, *p* < 0.05), more educated (*b* = 0.12, *p* < 0.05) and being of male gender (*b* = 1.27, *p* < 0.001) were more likely to belong to profile 1 compared to profile 3 (Self-directed researchers), while income did not significantly predict membership of profile 1.

For Profile 2 (Conventionalists) membership, education, political orientation, and gender were significant predictors. More educated individuals (*b* = 0.20, *p* < 0.05), individuals with more leftist political orientations (*b* = 0.35, *p* < 0.001), and men (*b* = 1.81, *p* < 0.001) were more likely to belong to this profile compared to profile 3. Age and income were not significant predictors of this profile.

**Figure 2:**
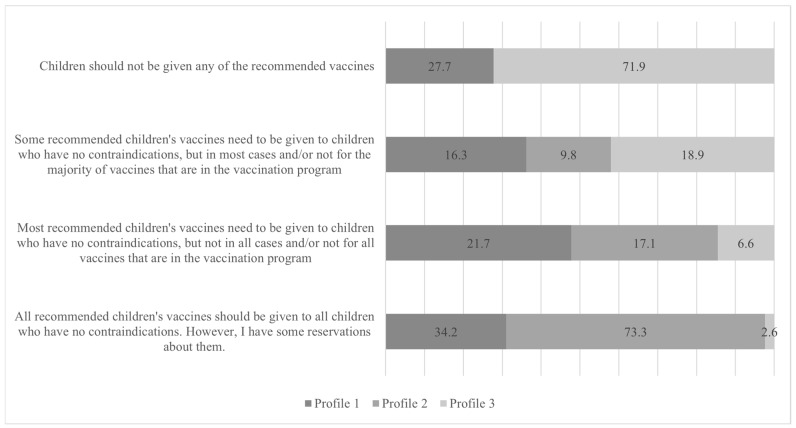
Differences in the intensity of vaccine hesitancy among profiles.

Figure 2 represents the results of the Chi-square test, which was used to examine the differences among profiles in their intensity of vaccine hesitancy (Cramer’s V = 0.45, *p* < 0.001). Profile 2 exhibited a weaker level of vaccine hesitancy, while profile 1 demonstrated a moderate level of hesitancy. Profile 3, on the other hand, revealed the greatest level of vaccine hesitation. 

We furthermore examined if the profiles differ in their attitudes towards vaccination and specific vaccines (Table 4). The former was investigated through attitudes towards efficacy, safety, and the importance of vaccines for one’s health. It was found that profile 1 consistently displayed more neutral attitudes towards vaccination, profile 2 showed the most positive attitudes, while profile 3 exhibited the most negative attitudes. Profiles 1 and 3 were the most skeptical about the safety of vaccines, (*M* = 2.56, *SD* = 1.28 and *M* = 1.24, *SD* = 0.63, respectively). Games–Howell post hoc comparisons indicated significant differences across all groups on all measured variables, as denoted by the superscript in the table.

Table 4 also shows differences in attitudes toward specific vaccines. Regarding the MMR vaccine, profile 2 showed the most positive attitudes, profile 1 displayed more neutral attitudes, and profile 3 expressed considerably negative attitudes towards this vaccine. The results also suggest that, while profiles 1 and 2 show differences in their degree of positive attitudes towards the MMR vaccine, both are more favorable when compared to profile 3. This pattern stayed consistent for the HPV and Seasonal Influenza vaccines; however, we observed a slight variation in the degree of difference in attitudes between the profiles across the different vaccines. This suggests that, while these groups can be characterized by their overall attitudes towards vaccination, the specific vaccine in question may influence the intensity of their attitudes. For instance, profile 1 scored 2.82 on the MMR vaccine and 2.31 on the Seasonal Influenza vaccine. Although both scores indicate a neutral stance, there is a slight difference between them. Similarly, for profile 3, the mean score for the HPV vaccine was 1.33, while, for the Seasonal Influenza vaccine, it was 1.13.

**Figure 3:**
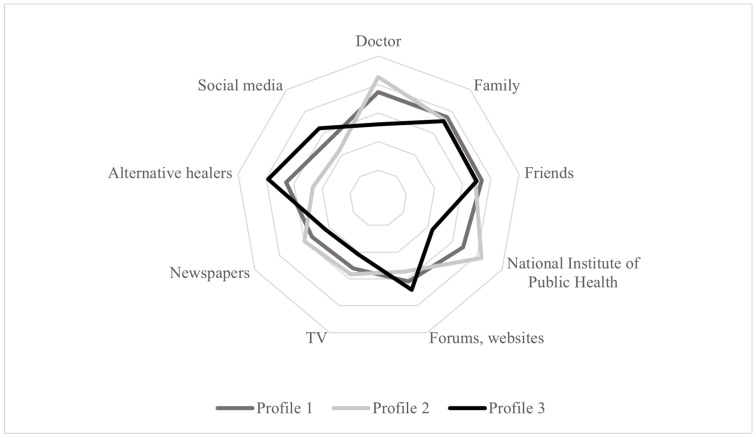
Importance of different information sources about vaccination across profiles.

Lastly, levels of importance of different sources of information about vaccines were examined across the three profiles (Figure 3). Profile 1 gave the highest level of importance to information obtained by doctors (*M* = 3.74, *SD* = 0.93), family (*M* = 3.74, *SD* = 0.90), friends (*M* = 3.68, *SD* = 0.81), and the National Institute of Public Health (*M* = 3.34, *SD* = 0.98). This profile also exhibited a tendency towards confidence in alternative healers (*M* = 3.28, *SD* = 0.95) but only gave a moderate level of importance to information in forums and websites (*M* = 3.09, *SD* = 0.92), social media (*M* = 2.68, *SD* = 0.98), and mainstream media, namely TV (*M* = 2.60, *SD* = 0.99) and newspapers (*M* = 2.68, *SD* = 0.96). 

Profile 2 gave the highest level of importance to information obtained from doctors (*M* = 4.27, *SD* = 0.87), followed by the National Institute of Public Health (*M* = 4.17, *SD* = 0.77). Surprisingly, the importance of family and friends as a source of information was slightly lower in this profile than in profile 1, with mean scores of 3.61 (*SD* = 0.74) and 3.46 (*SD* = 0.81), respectively. Online forums and websites, television, and newspapers were less important, with all mean scores falling below 3.00. Notably, of the three profiles, this group gave the lowest level of importance to information on social media (*M* = 2.17, *SD* = 0.95) and alternative healers (*M* = 2.34, *SD* = 0.99). 

Profile 3, on the other hand, gave the highest level of importance to alternative healers (*M* = 3.93, *SD* = 0.91), as well as a moderately high level of importance to forums and websites (*M* = 3.41, *SD* = 0.99) and social media (*M* = 3.22, *SD* = 1.08). With mean scores of 2.61 (*SD* = 1.25), 3.55 (*SD* = 1.10), and 3.50 (*SD* = 0.97), the importance of information about vaccines from doctors, family, and friends was lower. Among the three profiles, the importance of the National Institute of Public Health was the lowest (*M* = 2.18, *SD* = 1.22) and the importance of the mainstream media was likewise low (*M* = 2.08, *SD* = 1.11 for TV, and *M* = 2.16, *SD* = 1.05 for newspaper).

## 5. Discussion

The present study aimed to explore the complexity of vaccine hesitancy using latent profile analysis, which identified three distinct vaccine-hesitant profiles, namely Skeptics, Conventionalists, and Self-directed researchers. These profiles were examined through trust in the healthcare system, endorsement of complementary and alternative medicine, beliefs in conspiracy theories, reliance on personal research, and sociodemographic variables.

We identified three distinct profiles in our study. Skeptics demonstrated moderate trust in the healthcare system but had low endorsement of CAM and conspiracy theories and relied less on personal research. They were typically older, higher-educated men, with low overconfidence in their knowledge. Conventionalists showed the highest trust in the healthcare system, minimal reliance on personal research, and low overconfidence. This group included more educated individuals, men, and those with a leftist political orientation. Self-directed researchers, in contrast, had the highest distrust in the healthcare system, high endorsement of CAM and conspiracy theories, and high reliance on personal research. They also exhibited moderate overconfidence and the highest level of vaccine hesitancy. Furthermore, attitudes toward vaccines varied among the profiles. Conventionalists held positive views on vaccine efficacy, safety, and importance, Skeptics were neutral, and Self-directed researchers had the most negative attitudes. Trust in information sources also differed. Skeptics trusted doctors, family, friends, and the National Institute of Public Health but had moderate trust in alternative healers and lower trust in social media and mainstream media. Conventionalists prioritized doctors and the National Institute of Public Health, with less emphasis on family and friends, and had the lowest reliance on social media and alternative healers. Self-directed researchers, however, valued information from alternative healers the most, followed by online forums and social media. Information from doctors, family, and friends was less important, as was information about vaccines from the National Institute of Public Health and mainstream media. 

Vaccine hesitancy, especially more radical and opposing views of vaccines, were previously associated with trusting and obtaining information from social media [48], the Internet, and relatives [46]. In our study, these information sources were common amongst the “Self-directed researcher” category, which opposed vaccination the most. Interestingly, Conventionalists and, to some extent, Skeptics both expressed the importance of information from healthcare professionals, which was previously confirmed as an important predictor for positive attitudes [46]. This raises the interesting question of what role the intensity of beliefs and trust plays in vaccine hesitancy. 

Furthermore, our study, similar to previous LPA research, underscores the importance of sociodemographic characteristics in understanding vaccine hesitancy. Consistent with findings from Howard [4] and Hornsey et al. [31], the profiles highlight significant sociodemographic distinctions, such as education level and age. For instance, Conventionalists were generally higher-educated and older, aligning with Hornsey et al.’s [31] identification, who also found that the profile expressing the most positive attitudes also consisted of higher-educated individuals. In addition, our study confirms findings from Howard [4], who noted that conservative political orientation was most common among the most vaccine-hesitant profiles, and with Zhou et al. [5], who found that more liberal-leaning individuals were less hesitant in comparison to conservatives, who were in the profile which expressed the lowest vaccination intention. 

Drawing similarities or differences based on other constructs is more challenging, as the studies used different latent variables to identify profiles. However, we can partially compare our results with those of Lamot et al. [6], who used satisfaction with the healthcare system and endorsement of conspiracy theories as latent indicators. They found that individuals who were the most conspiratorial and distrustful were also more opposed to vaccination; this is consistent with our findings, where Self-directed researchers were the most conspiratorial and had the highest levels of vaccine-rejection. Similarly, Park et al. [60] reported the highest childhood vaccination intent within the profile of US mothers who were the least distrustful of medical institutions. In a study of Hong Kong nurses, the “skeptics” were the least likely to receive a vaccination and expressed the lowest levels of institutional trust [61]. Among Italian healthcare workers, Portoghese et al. [62] found that the highest levels of conspiracy beliefs were among the vaccine “rejector” and “hesitant” profiles. Among UK adults, the profile least likely to receive a vaccination was “social media users”, who, similarly to our findings, were most likely to obtain their health information from social media compared to other profiles [63]. 

Even though the present study contributes to theoretical and practical understanding of vaccine hesitancy, it has several limitations. First, the sample only included individuals from Slovenia, which may limit the generalization of the results to other countries. Future studies should use representative samples, including people from different regions, ages, genders, educational levels, and socioeconomic backgrounds. Moreover, cultural factors specific to Slovenia might have influenced these profiles. The Conventionalist profile consisted of a very small sample (*n* = 41), which may affect the robustness and reliability of findings related to this group. Additionally, the study relied on self-reported data, which are subject to biases such as social desirability and recall bias. Next, causality cannot be established due to the cross-sectional design of the study. Finally, the study focused on a limited number of variables, potentially overlooking other relevant determinants and traits. Future studies should include individuals’ characteristics, such as media use habits, the impact of social media use, religious affiliation, and past vaccination experiences.

Future research studies should delve more into the observed gradient in belief intensity between the profiles we detected, which suggests a promising avenue for further research. It could also further explore which profiles emerge in different cultural and societal contexts (i.e., replication of the numbers and characteristics of profiles in different cultural contexts). In addition, future research endeavors should consider long-term follow-up surveys to observe trends in vaccine hesitators, assess the effectiveness of interventions, and adjust strategies in time.

Our results also provide potential avenues for targeted interventions that are tailored to specific subgroups. Understanding the characteristics of vaccine-hesitant subgroups can help public health communication and campaigns develop more effective and targeted interventions. Skeptics, for example, who already express a certain level of trust in healthcare, may be most receptive to messages from medical professionals. Given that Skeptics are typically older, higher-educated men, interventions could focus on leveraging their existing trust in healthcare professionals and providing detailed, evidence-based information to address their specific concerns. Self-directed researchers may need a different approach that addresses their specific concerns and distrust of traditional healthcare systems. This group, characterized by high endorsement of CAM and conspiracy theories and moderate overconfidence, could benefit from interventions involving trusted community figures or alternative medicine experts who can bridge the gap between traditional and alternative medicine perspectives. In addition, reaching out to younger women in this group through online platforms and providing credible information that counteracts misinformation could be effective.

Conventionalists, who have the highest trust in the healthcare system and tend to include more educated individuals with a left-leaning political orientation, may respond well to public health messages that emphasize the collective benefits of vaccination. Campaigns could focus on reinforcing their positive views on the efficacy, safety, and importance of vaccines while leveraging their trust in physicians and public health institutions. Second, the effectiveness of targeted interventions for each specific subgroup could then also be examined, employing insights from our study. For instance, tailored communication strategies could be tested to determine which messages and messengers are the most effective in regard to changing attitudes and behaviors within each profile.

In conclusion, our findings not only further confirm the heterogeneous nature of vaccine-hesitant groups but also offer critical insights for public health interventions. By acknowledging the existence of distinct profiles within the vaccine-hesitant population, strategies can be tailored to address the nuanced beliefs and attitudes of these subgroups more effectively.

## Figures and Tables

**Table 1:** Model fit statistics.

Profiles	LL	AIC	BIC	SA-BIC	Entropy	LMR-*p*	BLRT-*p*	% Smallest Class
1	−2700.522	5421.044	5461.47	5429.737	/	/	/	/
2	−2406.051	4844.102	4908.784	4858.011	0.85	<0.001	<0.001	32.5
**3**	**−2324.466**	**4692.932**	**4781.87**	**4712.057**	**0.82**	**<0.001**	**<0.001**	**10.1**
4	−2290.096	4636.191	4749.385	4660.532	0.76	<0.001	<0.001	7.8
5	−2278.810	4625.62	4763.069	4655.176	0.77	0.394	0.013	6.6
6	−2268.700	4617.4	4779.105	4652.173	0.8	0.171	0.05	0.7

Note. LL = Log-Likelihood. AIC = Akaike Information Criterion. BIC = Bayesian Information Criterion. SA-BIC = Sample-Size Adjusted Bayesian Information Criterion. LMR-*p* = Lo–Mendell–Rubin Adjusted Likelihood Ratio Test. BLRT-*p* = Bootstrap Likelihood Ratio Test. Bolded is the model solutions that shows the best model fit.

**Table 2:** Profile Means on latent indicators.

	Profile 1		Profile 2		Profile 3			
	(*n* = 184)		(*n* = 41)		(*n* = 196)			
	*M*	*SD*	*M*	*SD*	*M*	*SD*	*F* (*df*)	η^2^
CAM ^a^	3.22 ^B,C^	0.55	2.22 ^C^	0.56	4.19	0.56	275.650 ***	0.62
							(2, 416)	
Conspiracy theories ^b^	3.4 ^B,C^	0.58	2.05 ^C^	0.56	4.33	0.53	318.506 ***	0.65
							(2, 145)	
Personal research ^b^	2.64 ^B,C^	0.7	1.71 ^C^	0.78	3.52	0.59	150.447 ***	0.5
							(2, 107.169)	
Trust in HCS ^b^	2.35 ^B,C^	0.57	3.46 ^C^	0.54	1.43	0.44	338.914 ***	0.66
							(2, 109.023)	
Overconfidence ^b^	1.95 ^C^	0.81	1.64 ^C^	0.88	2.66	0.92	41.247 ***	0.23
							(2, 109.02)	

Note. ^a^ ANOVA. ^b^ Welch’s ANOVA. Letters in superscripts indicate significant pairwise comparisons. CAM = complementary and alternative medicine. HCS = healthcare system. *** *p* < 0.001.

**Table 3:** Logistic regression of sociodemographic factors predicting profile membership.

		*B*	*SE*	*Wald*	*p*
Profile 1	Age	−0.03	0.01	4.357	0.04
	Gender (male)	1.27	0.36	12.624	<0.001
	Education	0.12	0.06	4.306	0.04
	Income	0	0	4.945	0.03
	Political orientation	−0.01	0.06	0.01	0.92
Profile 2	Age	−0.00	0.02	0.025	0.87
	Gender (male)	1.81	0.51	12.752	<0.001
	Education	0.2	0.1	3.86	0.05
	Income	0	0	2.015	0.16
	Political orientation	−0.35	0.1	13.382	<0.001

Note. Profile 3 (Self-directed researchers) is the reference category. *B* = unstandardized beta; *SE* = standard error; *Wald* = Wald statistical test.

**Table 4:** Differences between profiles in vaccine attitudes.

		Profile 1	Profile 2	Profile 3		
		(A)	(B)	(C)		
		*M* (*SD*)	*M* (*SD*)	*M* (*SD*)	*F* (*df*)	η^2^
Attitudes towards vaccination	Efficacy	3.00 (1.25) ^B,C^	4.29 (0.90) ^C^	1.63 (1.00)	168.455 ***	0.39
					(2, 120.527)	
	Safety	2.56 (1.28) ^B,C^	3.93 (0.78) ^C^	1.24 (0.63)	257.162 ***	0.44
					(2, 108.588)	
	Importance	2.79 (1.32) ^B,C^	4.29 (0.95) ^C^	1.42 (0.80)	201.427 ***	0.43
					(2, 110.484)	
Attitudes toward specific vaccines	MMR	2.82 (1.39) ^B,C^	4.29 (0.89) ^C^	1.38 (0.86)	189.177 ***	0.42
					(2, 97.113)	
	HPV	2.56 (1.29) ^B,C^	3.58 (1.15) ^C^	1.33 (0.81)	91.483 ***	0.26
					(2, 78.064)	
	Seasonal Influenza	2.31 (1.17) ^B,C^	3.50 (1.11) ^C^	1.13 (0.51)	148.688 ***	0.43
					(2, 93.928)	

Note. Presented is Welch’s ANOVA and Games–Howell post hoc test, due to non-homogeneity of variances and unequal sizes of groups. Letters in superscripts indicate significant pairwise comparisons. *** *p* < 0.001.

## Data Availability

The data are available upon reasonable request to the authors.

## References

[1] MacDonald N.E. (2015). Vaccine hesitancy: Definition, scope and determinants. Vaccine.

[2] Allington D., McAndrew S., Moxham-Hall V., Duffy B. (2021). Coronavirus conspiracy suspicions, general vaccine attitudes, trust and coronavirus information source as predictors of vaccine hesitancy among UK residents during the COVID-19 pandemic. Psychol. Med..

[3] Hwang S.E., Kim W.-H., Heo J. (2022). Socio-demographic, psychological, and experiential predictors of COVID-19 vaccine hesitancy in South Korea, October–December 2020. Hum. Vaccin. Immunother..

[4] Howard M.C. (2023). Integrating the person-centered approach with the study of vaccine hesitancy: Applying latent profile analysis to identify vaccine hesitancy subpopulations and assess their relations with correlates and vaccination outcomes. Vaccine.

[5] Zhou Y., Li R., Shen L. (2023). Psychological profiles of COVID vaccine-hesitant individuals and implications for vaccine message design strategies. Vaccine X.

[6] Lamot M., Kerman K., Kirbiš A. (2022). Distrustful, Dissatisfied, and Conspiratorial: A Latent Profile Analysis of COVID-19 Vaccination Rejection. Int. J. Environ. Res. Public Health.

[7] Larson H.J., Jarrett C., Eckersberger E., Smith D.M.D., Paterson P. (2014). Understanding vaccine hesitancy around vaccines and vaccination from a global perspective: A systematic review of published literature, 2007–2012. Vaccine.

[8] Toshkov D. (2023). What accounts for the variation in COVID-19 vaccine hesitancy in Eastern, Southern and Western Europe?. Vaccine.

[9] Browne M., Thomson P., Rockloff M.J., Pennycook G. (2015). Going against the Herd: Psychological and Cultural Factors Underlying the ‘Vaccination Confidence Gap’. PLoS ONE.

[10] Hornsey M.J., Lobera J., Díaz-Catalán C. (2020). Vaccine hesitancy is strongly associated with distrust of conventional medicine, and only weakly associated with trust in alternative medicine. Soc. Sci. Med..

[11] Ahorsu D.K., Lin C.-Y., Yahaghai R., Alimoradi Z., Broström A., Griffiths M.D., Pakpour A.H. (2022). The mediational role of trust in the healthcare system in the association between generalized trust and willingness to get COVID-19 vaccination in Iran. Hum. Vaccin. Immunother..

[12] Özer Ö., Budak F., Alp S. (2023). Is Vaccine Hesitancy Affected by Distrust in the Healthcare System? A Study in Turkish Population. Soc. Work. Public. Health.

[13] Jennings W., Stoker G., Bunting H., Valgarðsson V.O., Gaskell J., Devine D., McKay L., Mills M.C. (2021). Lack of Trust, Conspiracy Beliefs, and Social Media Use Predict COVID-19 Vaccine Hesitancy. Vaccines.

[14] Nazlı Ş.B., Yığman F., Sevindik M., Deniz Özturan D. (2022). Psychological factors affecting COVID-19 vaccine hesitancy. Ir. J. Med. Sci..

[15] Hoffman B.L., Felter E.M., Chu K.-H., Shensa A., Hermann C., Wolynn T., Williams D., Primack B.A. (2019). It’s not all about autism: The emerging landscape of anti-vaccination sentiment on Facebook. Vaccine.

[16] Kata A. (2012). Anti-vaccine activists, Web 2.0, and the postmodern paradigm--an overview of tactics and tropes used online by the anti-vaccination movement. Vaccine.

[17] Vrdelja M., Kraigher A., Verčič D., Kropivnik S. (2018). The growing vaccine hesitancy: Exploring the influence of the internet. Eur. J. Public Health.

[18] Motta M., Callaghan T., Sylvester S. (2018). Knowing less but presuming more: Dunning-Kruger effects and the endorsement of anti-vaccine policy attitudes. Soc. Sci. Med..

[19] Morales D.X., Beltran T.F., Morales S.A. (2022). Gender, socioeconomic status, and COVID-19 vaccine hesitancy in the US: An intersectionality approach. Sociol. Health Illn..

[20] Fadhel F.H. (2021). Vaccine hesitancy and acceptance: An examination of predictive factors in COVID-19 vaccination in Saudi Arabia. Health Promot. Int..

[21] Troiano G., Nardi A. (2021). Vaccine hesitancy in the era of COVID-19. Public Health.

[22] Schwarzinger M., Watson V., Arwidson P., Alla F., Luchini S. (2021). COVID-19 vaccine hesitancy in a representative working-age population in France: A survey experiment based on vaccine characteristics. Lancet Public Health.

[23] Willis D.E., Andersen J.A., Bryant-Moore K., Selig J.P., Long C.R., Felix H.C., Curran G.M., McElfish P.A. (2021). COVID-19 vaccine hesitancy: Race/ethnicity, trust, and fear. Clin. Transl. Sci..

[24] Bocquier A., Ward J., Raude J., Peretti-Watel P., Verger P. (2017). Socioeconomic differences in childhood vaccination in developed countries: A systematic review of quantitative studies. Expert. Rev. Vaccines.

[25] Galarce E.M., Minsky S., Viswanath K. (2011). Socioeconomic status, demographics, beliefs and A(H1N1) vaccine uptake in the United States. Vaccine.

[26] Robertson E., Reeve K.S., Niedzwiedz C.L., Moore J., Blake M., Green M., Katikireddi S.V., Benzeval M.J. (2021). Predictors of COVID-19 vaccine hesitancy in the UK household longitudinal study. Brain Behav. Immun..

[27] Fridman A., Gershon R., Gneezy A. (2021). COVID-19 and vaccine hesitancy: A longitudinal study. PLoS ONE.

[28] Howard M.C. (2022). Investigating the Relation of Political Orientation and Vaccination Outcomes: Identifying the Roles of Political Ideology, Party Affiliation, and Vaccine Hesitancy. Psychol. Rep..

[29] Stroope S., Kroeger R.A., Williams C.E., Baker J.O. (2021). Sociodemographic correlates of vaccine hesitancy in the United States and the mediating role of beliefs about governmental conspiracies. Soc. Sci. Q..

[30] Pires C. (2022). Global Predictors of COVID-19 Vaccine Hesitancy: A Systematic Review. Vaccines.

[31] Hornsey M.J., Edwards M., Lobera J., Díaz-Catalán C., Barlow F.K. (2021). Resolving the small-pockets problem helps clarify the role of education and political ideology in shaping vaccine scepticism. Br. J. Psychol..

[32] Gravelle T.B., Phillips J.B., Reifler J., Scotto T.J. (2022). Estimating the size of “anti-vax” and vaccine hesitant populations in the US, UK, and Canada: Comparative latent class modeling of vaccine attitudes. Hum. Vaccin. Immunother..

[33] Kricorian K., Civen R., Equils O. (2022). COVID-19 vaccine hesitancy: Misinformation and perceptions of vaccine safety. Hum. Vaccin. Immunother..

[34] Edwards K.M., Hackell J.M. (2016). Countering Vaccine Hesitancy. Pediatrics.

[35] Chamberlain A.T., Seib K., Ault K.A., Orenstein W.A., Frew P.M., Malik F., Cortés M., Cota P., Whitney E.A.S., Flowers L.C. (2015). Factors Associated with Intention to Receive Influenza and Tetanus, Diphtheria, and Acellular Pertussis (Tdap) Vaccines during Pregnancy: A Focus on Vaccine Hesitancy and Perceptions of Disease Severity and Vaccine Safety. PLoS Curr..

[36] Suryadevara M., Handel A., Bonville C.A., Cibula D.A., Domachowske J.B. (2015). Pediatric provider vaccine hesitancy: An under-recognized obstacle to immunizing children. Vaccine.

[37] Roberts J.R., Thompson D., Rogacki B., Hale J.J., Jacobson R.M., Opel D.J., Darden P.M. (2015). Vaccine hesitancy among parents of adolescents and its association with vaccine uptake. Vaccine.

[38] Soares P., Rocha J.V., Moniz M., Gama A., Laires P.A., Pedro A.R., Dias S., Leite A., Nunes C. (2021). Factors Associated with COVID-19 Vaccine Hesitancy. Vaccines.

[39] Lucia V.C., Kelekar A., Afonso N.M. (2021). COVID-19 vaccine hesitancy among medical students. J. Public Health.

[40] Murphy J., Vallières F., Bentall R.P., Shevlin M., McBride O., Hartman T.K., McKay R., Bennett K., Mason L., Gibson-Miller J. (2021). Psychological characteristics associated with COVID-19 vaccine hesitancy and resistance in Ireland and the United Kingdom. Nat. Commun..

[41] Khubchandani J., Sharma S., Price J.H., Wiblishauser M.J., Sharma M., Webb F.J. (2021). COVID-19 Vaccination Hesitancy in the United States: A Rapid National Assessment. J. Commun. Health.

[42] Aw J., Seng J.J.B., Seah S.S.Y., Low L.L. (2021). COVID-19 Vaccine Hesitancy-A Scoping Review of Literature in High-Income Countries. Vaccines.

[43] Dubé E., Laberge C., Guay M., Bramadat P., Roy R., Bettinger J. (2013). Vaccine hesitancy: An overview. Hum. Vaccin. Immunother..

[44] Karafillakis E., Simas C., Jarrett C., Verger P., Peretti-Watel P., Dib F., de Angelis S., Takacs J., Ali K.A., Pastore Celentano L. (2019). HPV vaccination in a context of public mistrust and uncertainty: A systematic literature review of determinants of HPV vaccine hesitancy in Europe. Hum. Vaccin. Immunother..

[45] Weiss C., Schröpfer D., Merten S. (2016). Parental attitudes towards measles vaccination in the canton of Aargau, Switzerland: A latent class analysis. BMC Infect. Dis..

[46] Charron J., Gautier A., Jestin C. (2020). Influence of information sources on vaccine hesitancy and practices. Med. Mal. Infect..

[47] Puri N., Coomes E.A., Haghbayan H., Gunaratne K. (2020). Social media and vaccine hesitancy: New updates for the era of COVID-19 and globalized infectious diseases. Hum. Vaccin. Immunother..

[48] Reno C., Maietti E., Di Valerio Z., Montalti M., Fantini M.P., Gori D. (2021). Vaccine Hesitancy towards COVID-19 Vaccination: Investigating the Role of Information Sources through a Mediation Analysis. Infect. Dis. Rep..

[49] Thornton L., Batterham P.J., Fassnacht D.B., Kay-Lambkin F., Calear A.L., Hunt S. (2016). Recruiting for health, medical or psychosocial research using Facebook: Systematic review. Internet Interv..

[50] King D.B., O’Rourke N., DeLongis A. (2014). Social media recruitment and online data collection: A beginner’s guide and best practices for accessing low-prevalence and hard-to-reach populations. Can. Psychol..

[51] Hyland M.E., Lewith G.T., Westoby C. (2003). Developing a measure of attitudes: The holistic complementary and alternative medicine questionnaire. Complement. Ther. Med..

[52] Brotherton R., French C.C., Pickering A.D. (2013). Measuring belief in conspiracy theories: The generic conspiracist beliefs scale. Front. Psychol..

[53] Bianco A., Mascaro V., Zucco R., Pavia M. (2019). Parent perspectives on childhood vaccination: How to deal with vaccine hesitancy and refusal?. Vaccine.

[54] Rozbroj T., Lyons A., Lucke J. (2019). Psychosocial and demographic characteristics relating to vaccine attitudes in Australia. Patient Educ. Couns..

[55] Casiday R., Cresswell T., Wilson D., Panter-Brick C. (2006). A survey of UK parental attitudes to the MMR vaccine and trust in medical authority. Vaccine.

[56] Navalpakam A., Dany M., Hajj Hussein I. (2016). Behavioral Perceptions of Oakland University Female College Students towards Human Papillomavirus Vaccination. PLoS ONE.

[57] Ferguson S.L., Moore E.W.G., Hull D.M. (2020). Finding latent groups in observed data: A primer on latent profile analysis in Mplus for applied researchers. Int. J. Behav. Dev..

[58] Spurk D., Hirschi A., Wang M., Valero D., Kauffeld S. (2020). Latent profile analysis: A review and “how to” guide of its application within vocational behavior research. J. Vocat. Behav..

[59] Tein J.-Y., Coxe S., Cham H. (2013). Statistical Power to Detect the Correct Number of Classes in Latent Profile Analysis. Struct. Equ. Model..

[60] Park Y.W., Bragard E., Madhivanan P., Fisher C.B. (2024). A Latent Profile Analysis of COVID-19 and Influenza Vaccine Hesitancy among Economically Marginalized Hispanic Mothers of Children under Five Years of Age in the US. J. Racial Ethn. Health Disparities.

[61] Leung C.L.K., Li K.-K., Wei V.W., Tang A., Wong S.Y.S., Lee S.S., Kwok K.O. (2022). Profiling vaccine believers and skeptics in nurses: A latent profile analysis. Int. J. Nurs. Stud..

[62] Portoghese I., Siddi M., Chessa L., Costanzo G., Garcia-Larsen V., Perra A., Littera R., Sambugaro G., Del Giacco S., Campagna M. (2023). COVID-19 Vaccine Hesitancy among Italian Healthcare Workers: Latent Profiles and Their Relationships to Predictors and Outcome. Vaccines.

[63] Colville S., Lockey S., Gillespie N., Jane Kelly S. (2024). Compliance with COVID-19 preventative health measures in the United Kingdom: A latent profile analysis. Health Promot. Int..

